# Copula-based modeling and simulation of 3D systems of curved fibers by isolating intrinsic fiber properties and external effects

**DOI:** 10.1038/s41598-023-46644-5

**Published:** 2023-11-08

**Authors:** Matthias Weber, Andreas Grießer, Dennis Mosbach, Erik Glatt, Andreas Wiegmann, Volker Schmidt

**Affiliations:** 1 Institute of Stochastics, Ulm University, Helmholtzstraße 18, 89069 Ulm, Germany; 2 Math2Market GmbH, Richard-Wagner-Straße 1, 67655 Kaiserslautern, Germany

**Keywords:** Applied mathematics, Coarse-grained models, Porous materials

## Abstract

In this paper we lay the foundation for data-driven 3D analysis of virtual fiber systems with respect to their microstructure and functionality. In particular, we develop a stochastic 3D model for systems of curved fibers similar to nonwovens, which is fitted to tomographic image data. By systematic variations of model parameters, efficient computer-based scenario analyses can be performed to get a deeper insight how effective properties of this type of functional materials depend on their 3D microstructure. In a first step, we consider single fibers as polygonal tracks which can be modeled by a third-order Markov chain. For constructing the transition function of the Markov chain, we formalize the intuitive notions of intrinsic fiber properties and external effects and build a copula-based transition function such that both aspects can be varied independently. Using this single-fiber model, in a second step we derive a model for the entire fiber system observed in a bounded sampling window and fit it to two different 3D datasets of nonwovens measured by CT imaging. Considering various geometric descriptors of the 3D microstructure related to effective properties of the pore space, we evaluate the goodness of model fit by comparing geometric descriptors of the 3D morphology of model realizations with those of tomographic image data.

## Introduction

A wide range of applications from fuel cell technology^1^, hygiene products^2^ and filtration^3^ heavily depend on the usage of fiber-based materials like nonwovens. Here, various properties of nonwovens like diffusivity or wettability must be tailored to meet specific needs. Improving on these properties by means of traditional manufacturing and testing is expensive and time-consuming. However, when the microstructure of a nonwoven is known, numerical methods can be used for in-silico investigation of effective properties. Combined with a stochastic microstructure model, this can be used to perform virtual materials testing by creating and investigating realistic structures which yet have not been considered before. By this approach, insight into the relationship between microstructure geometry and effective properties can be obtained at a low cost which can then be used to streamline the procedure for developing materials with enhanced properties^4–6^.

Various stochastic microstructure models for nonwovens have been proposed in the literature which are used for different purposes like filter media^7–10^, gas-diffusion layers in fuel cells^1,11,12^ and general applications^13–17^, while other models are concerned with specific fiber properties like orientation and distance^18,19^. In^20^, we recently developed a stochastic model for creating single fibers similar to those seen in various nonwoven materials. This model was based on representing fibers using the so-called Frenet-Serret formulas, but did not include any information about the global appearance of the fiber system. In the present paper, we adapt this model to account for what one might intuitively describe as intrinsic properties of fibers as well as external effects acting upon fibers. Roughly speaking, we formalize these notions by considering all those properties as intrinsic which do not affect the global appearance of fibers, where we consider as external effects only information about the global orientation of parts of fibers based on the distance of the fiber segments to the upper and lower boundaries of the material. Combining both intrinsic properties and external effects allows for the application of the developed model to describe entire fiber systems.

**Figure 1:**
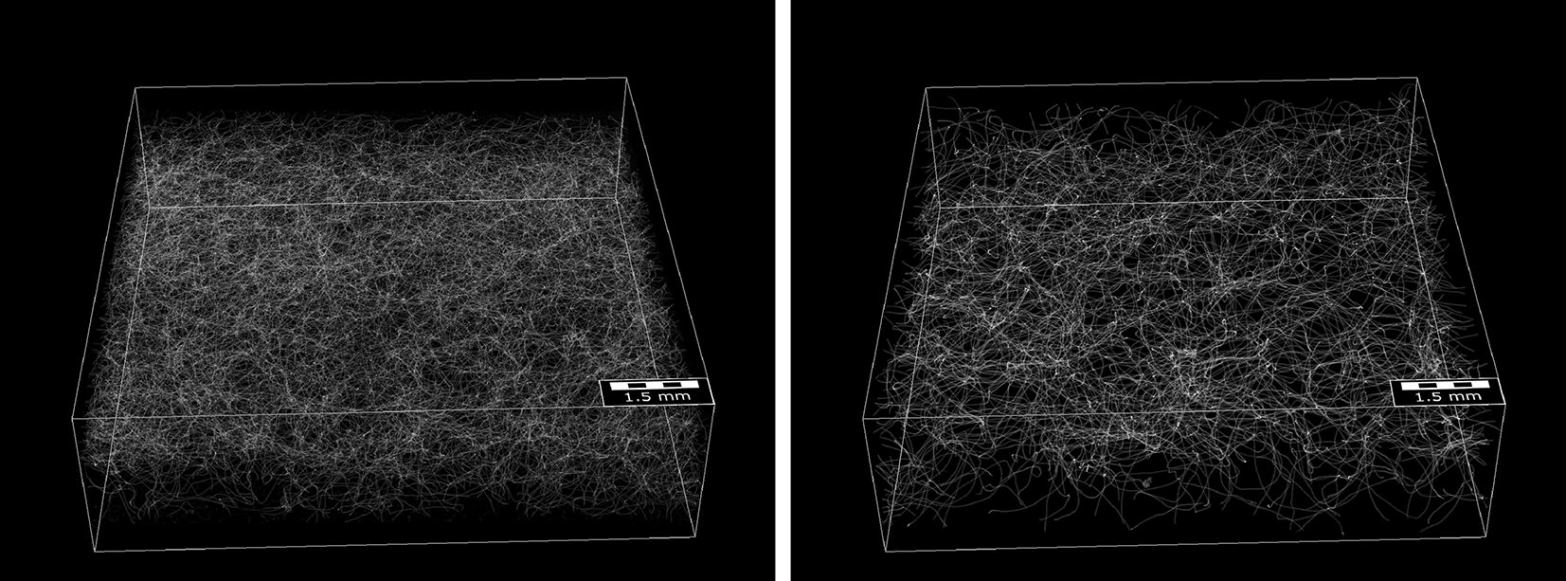
3D view of the gray value image of Sample 1 (left) and Sample 2 (right).

**Figure 2:**
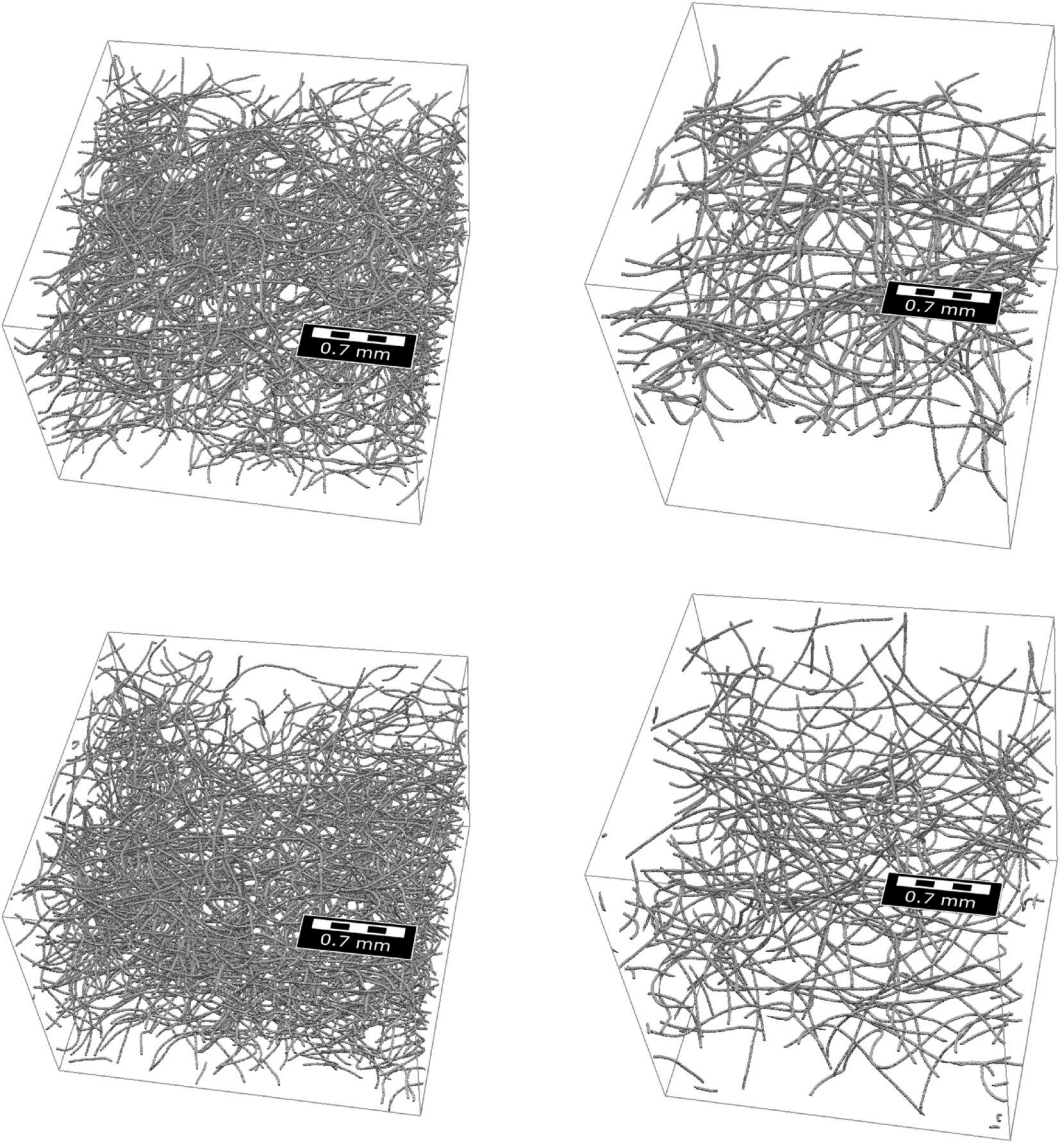
Cutouts of measured (top) and simulated (bottom) fiber systems for Sample 1 (left) and Sample 2 (right). See also Fig. 1.

We assume that each fiber can be considered as a third-order Markov chain^21^ and model the transition function as a product of two (conditional) univariate probability densities, one of which represents the intrinsic properties of fibers and the other one represents the external effects. The underlying (unconditional) bi- and trivariate probability densities are in turn modeled by means of copulas^22,23^, which provide a powerful tool for stochastic modeling of non-Gaussian correlated vector data. Together with a simple model for the generation of initial points, this Markov chain constitutes the basis of a model for entire fiber systems which we fit to two different 3D datasets of nonwovens measured by CT imaging, see Fig. 1, and processed using a convolutional neural network^24,25^ to extract the center-lines of fibers. Considering various geometric descriptors of the 3D microstructure related to effective properties of the pore space, we evaluate the goodness of model fit by comparing geometric descriptors of the 3D morphology of model realizations with those of tomographic image data. For the two different material samples considered in this paper, Fig. 2 shows both segmented 3D images of measured fiber systems along with realizations of the respective fitted models. The overall visual impression of Fig. 2 is that measured and simulated fiber systems are quite similar for each of the two samples, but rather different between the two samples. This qualitative assessment will be specified later in the paper.

The rest of the paper is organized as follows. We first describe the methods used for constructing and validating the model as well as the underlying measured data. In particular, we describe the stochastic 3D model for single curved fibers and entire fiber systems, which is fitted to tomographic image data. Furthermore, we evaluate the goodness of model fit by comparing geometric descriptors of the 3D morphology of model realizations with those of CT data. Finally, we discuss the obtained results and outline possible further research.

## Materials and methods

### Description of nonwoven material

We investigated two samples of nonwoven materials in this paper. The size of Sample 1 is 11.2 mm $\times$ 11.2 mm $\times$ 1.9 mm and that of Sample 2 is 10.8 mm $\times$ 10.8 mm $\times$ 2.1 mm. Sample 1 consists of a single type of circular Polyethylen-Polyethylenterephthalat (PE-PET) fibers. The material has a solid volume fraction of 1.4% and an average fiber diameter of 18.12 micron. Sample 2 consists of a single type of circular Polyethylenterephthalat (PET) fibers. Its solid volume fraction of is 0.71% and fibers have an average diameter of 22.65 micron. Both samples consist of staple fibers with lengths of a few millimeters and monodisperse diameters.

These nonwovens were specifically created to investigate the curvature of fiber systems. The usual air bonding step was omitted to simplify the identification of individual fibers. Similar materials are commonly used in hygiene products, making the results of this research relevant for such applications. By employing these unique nonwovens, we can explore the inherent curvature properties of fiber systems and gain valuable insights on how to model these types of fiber systems.

### Data acquisition

The two nonwoven materials were scanned in a Scanco MicroCT50 micro-CT scanner at a resolution of 2 micron and an energy of 45KeV. Overall 3600 projections were taken using Off-Axis scanning. Each projection was integrated for 4.5 seconds. For reconstruction the standard Feldkamp-Davis-Kress (FDK)^26^ algorithm was used. A visualization of the gray value images is shown in Fig. 1. We use GeoDict^27^ to perform the necessary image processing steps and segmenting the scans into fibers and background.

To analyze the fiber system within the nonwoven, the individual fibers are identified and their geometry is analyzed. This is done with the FiberFind module of GeoDict. The algorithm used in that module is described in^25^. As discussed there, this method provides good results for nonwoven structures, especially those that are used in hygiene products. It uses a neural network to identify the center line of each fiber in the segmented micro-CT scan. This neural network was pre-trained on artificial models of nonwoven structures to avoid manually labeling of training data and is included in GeoDict. As a neural network architecture, a 3D U-Net^28^ is used. The center lines of the fibers labeled by the neural network are utilized to construct a graph representation of the complete fiber network. In the graph, endpoints are investigated to check if they are true fiber endpoints. If fiber fragments are found that are well aligned and the endpoints are close to each other the separation is considered an error and the fragments are reconnected. After these corrections, each connected component of the graph represents exactly one fiber. The connected components are then used to create analytic representations of the fibers, where each fiber is represented by a list of piece-wise linear segments and respective diameters. The diameters are determined by averaging the center-line values of an euclidean distance transform inside the fibers. Using these analytic representation, various properties of the fibers, including orientation, curvature, and length, can easily be computed.

### Model for nonwoven materials

In the following, we develop a stochastic microstructure model for curved fiber systems. This model is based on representing fibers as polygonal tracks which are then modeled by a third-order Markov chain. We choose a specific approach to model the transition function of this Markov chain and select and fit the underlying parametric distribution families in a subsequent step. Finally, we validate the model by simulating artificial fiber systems and statistically comparing them to data of measured fiber systems.

#### Third-order Markov chain for modeling the trajectories of single fibers

To describe our model for entire fiber systems, we start by modeling single curved fibers. For this purpose, we adapt the stochastic 3D model previously proposed in^20^. We assume that fibers exhibit a circular cross-section with a constant radius $\delta > 0$ and assume that the center-lines of fibers can be modeled as finite cutouts of an infinite random polygonal track, i.e., as parts of a sequence of random vectors $\ldots , P_{-2}, P_{-1}, P_0, P_1, P_2, \ldots :\Omega \rightarrow \mathbb {R}^3$ such that $|P_{n+1} - P_n| = c$ for each integer $n \in \mathbb {Z}=\{\ldots ,-1,0,1,\ldots \}$ for some constant segment length $c \in \mathbb {R}$, where $|\cdot |$ denotes the Euclidean norm. Choosing a constant segment length hugely simplifies the definition of our model and by appropriately choosing *c*, all fibers observed in measured data can be represented with reasonable accuracy. Moreover, we assume that the random sequence $\{P_n, n \in \mathbb {Z}\}$ satisfies the following three conditions.

(i) $\{P_n, n \in \mathbb {Z}\}$ forms a third-order Markov chain^21^, i.e., for each $n \in \mathbb {Z}$ and any finite subset $I \subset \mathbb {Z}$ with $\max \{i:i\in I\} < n-3$, the random vector $P_n$ is conditionally independent of the random vectors $\{P_i, i \in I\}$, under the condition that the values of $P_{n-3}, P_{n-2}, P_{n-1}$ are given. Note that this assumption is justified by the statistical analysis of measured and simulated image data performed later on in this paper.
(ii) We furthermore assume that the Markov chain $\{P_n, n \in \mathbb {Z}\}$ is stationary, i.e., for any finite subset $I \subset \mathbb {Z}$ and for each $k\ge 1$, the distributions of the sequences $\{P_i, i \in I\}$ and $\{P_{i+k}, i \in I\}$ coincide. The latter assumption is motivated by the fact that from a statistical point of view the morphological properties of fibers do not change along their trajectories.
(iii) Finally, we assume that the stationary Markov chain $\{P_n, n \in \mathbb {Z}\}$ is reversible, i.e., for each $k\ge 1$, the distributions of $(P_0,P_1,\ldots ,P_k)$ and $(P_0,P_{-1},\ldots ,P_{-k})$ coincide. This assumption is motivated by the circumstance that the statistical behavior of fiber trajectories is the same regardless of whether we traverse them forwards or backwards.

The conditions (i) - (iii) stated above imply that the distribution of the random sequence $\{P_n, n \in \mathbb {Z}\}$ is fully characterized by the joint distribution of the four random vectors $P_1,P_2,P_3,P_4$. In the following we assume that this distribution possesses a probability density, which will be denoted by $f_{P_1,P_2,P_3,P_4}:\mathbb {R}^{4\cdot 3}\rightarrow [0,\infty )$. Thus, modeling the random sequence $\{P_n, n \in \mathbb {Z}\}$ boils down to modeling the probability density $f_{P_1,P_2,P_3,P_4}$ of $(P_1,P_2,P_3,P_4)$.

#### Isolating intrinsic fiber properties and external effects

Intuitively, one may be inclined to distinguish between intrinsic properties of the fibers and external effects, which may influence the morphological properties of fibers. More formally, we will model the probability density $f_{P_1,P_2,P_3,P_4}$ by a product of three probability densities of independent random vectors which represent the position and orientation of fibers, intrinsic properties of fibers and external effects, respectively. For this purpose, we are going to denote the *x*-, *y*- and *z*-coordinates of $P_n$ by $X_n$, $Y_n$ and $Z_n$ for each $n\in \mathbb {Z}$ and use a similar notation for a realization $p_n$ of the random vector $P_n=(X_n,Y_n,Z_n)$, i.e., $p_n =(x_n, y_n, z_n) \in \mathbb {R}^3$.

As mentioned above, the model for $f_{P_1,P_2,P_3,P_4}$ consists of three components. First we consider the location of $P_1$ and the orientation of the vector $(P_1,P_2)$, which can be expressed in terms the angle between $P_2 - P_1$ and the unit vector (1, 0) (pointing along the *x*-axis), where, for now, we set aside the *z*-coordinates of $P_1$ and $P_2$. This is, we model the joint density $f_{X_1, Y_1, A_0}:\mathbb {R}^2\times [-\pi ,\pi ]\rightarrow [0,\infty )$ of $X_1, Y_1$ and $A_0 = {{\,\textrm{ang}\,}}((X_2, Y_2) - (X_1, Y_1))$, where ${{\,\textrm{ang}\,}}(p)$ denotes the angle between $p = (p_x, p_y) \in \mathbb {R}^2$ and the unit vector (1, 0). We define ${{\,\textrm{ang}\,}}(p) < 0$ if *p* is below the *x*-axis, and ${{\,\textrm{ang}\,}}(p) \ge 0$ otherwise. Note that in computer science, the mapping $p\mapsto {{\,\textrm{ang}\,}}(p)$ is often referred to as the four-quadrant inverse tangent (denoted by ${{\,\textrm{atan2}\,}}$), i.e., ${{\,\textrm{ang}\,}}(p) = {{\,\textrm{atan2}\,}}(p_y, p_x)$. Note that, while the distribution of $A_0$ observed in measured image data is not exactly uniform, for simplicity of the model we assume $A_0$ to be uniformly distributed on the interval $(-\pi , \pi )$.

Then, we model the behavior of the fibers in *z*-direction, which captures the most important external effects for the data considered in the present paper. As the fiber systems are bounded in *z*-direction by some (lower and upper) parallel planes, fibers should not pierce through these planes, but, instead, be redirected towards the interior between the planes. We will model these external effects by appropriately modeling the *z*-coordinates $Z_1,Z_2,Z_3,Z_4$ of $P_1,P_2,P_3, P_4$. In particular, we assume that the random sequence $\{Z_n, n \in \mathbb {Z}\}$ forms a stationary second-order Markov chain. This implies that modeling the joint density $f_{Z_1, Z_2, Z_3, Z_4}:\mathbb {R}^4\rightarrow [0,\infty )$ of $Z_1,Z_2,Z_3,Z_4$ only requires modeling the joint density $f_{Z_1, Z_2, Z_3}:\mathbb {R}^3\rightarrow [0,\infty )$ of $\bigl (Z_1, Z_2, Z_3\bigr )$, because it holds that

1 $$\begin{aligned} f_{Z_1, Z_2, Z_3, Z_4}(z_1, z_2, z_3, z_4) = f_{Z_1, Z_2} \bigl (z_1, z_2\bigr ) f_{Z_3 \mid Z_1 = z_1, Z_2=z_2} \bigl (z_3\bigr ) f_{Z_3 \mid Z_1=z_2, Z_2 = z_3} \bigl (z_4\bigr ) \end{aligned}$$

for any $z_1,z_2,z_3,z_4\in \mathbb {R}$, where $f_{Z_3 \mid Z_1 = z_i, Z_2 = z_{i+1}}:\mathbb {R}\rightarrow [0,\infty )$ is the conditional density of $Z_3$ under the condition that $Z_1=z_i$ and $Z_2=z_{i+1}$ for $i=2,3$ which is given by

$$\begin{aligned} f_{Z_3 \mid Z_1 = z_i, Z_2 = z_{i+1}}(z_j)=\frac{f_{Z_1,Z_2,Z_3}(z_i,z_{i+1},z_j)}{\int _{-\infty }^{\infty }f_{Z_1,Z_2,Z_3}(z_i,z_{i+1},z) \textrm{d}z}\qquad \text{ for } \text{ any } z_i,z_{i+1},z_j\in \mathbb {R}. \end{aligned}$$

It is clear that, instead of modeling $f_{Z_1, Z_2, Z_3}$, we may equivalently choose to model the joint density of $Z_1, Z_2 - Z_1$ and $Z_3 - Z_2$ which we will use in the following.

Finally, to capture intrinsic fiber properties, we consider the curvature of the fibers which is directly related to the angles between neighboring fiber segments $P_n - P_{n-1}$ and $P_{n+1} - P_n$. To account for features like persistence of curvature, we do not model these angles independently from each other, but by means of another stationary (first-order) Markov chain. More precisely, as we need to keep these angles independent of the *z*-coordinates $Z_1,Z_2,Z_3,Z_4$ of $P_1,P_2,P_3, P_4$, we only model the angles between the projections of consecutive fiber segments onto the *x*-*y*-plane. Thus, we consider the angles $A = \mathcal {A}((X_3, Y_3) - (X_2, Y_2), (X_2, Y_2) - (X_1, Y_1))$ and $B = \mathcal {A}((X_4, Y_4) - (X_3, Y_3), (X_3, Y_3) - (X_2, Y_2))$. Here, $\mathcal {A} :\mathbb {R}^2 \times \mathbb {R}^2 \rightarrow [-\pi , \pi ]$ denotes the (signed) angle between two vectors in the *x*-*y*-plane, i.e., $\mathcal {A}(s_1, s_2) = {{\,\textrm{ang}\,}}\bigl (s_2\bigr ) - {{\,\textrm{ang}\,}}\bigl (s_1\bigr )$ for any $s_1,s_2\in \mathbb {R}^2$, where ${{\,\textrm{ang}\,}}(s_i)$ is defined as described above. The joint density $f_{A, B}:[-\pi ,\pi ]^2\rightarrow [0,\infty )$ of *A* and *B* will be the third building stone in our model for $f_{P_1,P_2,P_3,P_4}$.

In summary, we model the probability density $f_{P_1,P_2,P_3,P_4}$ of $P_1, \ldots , P_4$ as product of the probability densities $f_{X_1, Y_1, A_0}$, $f_{Z_1, Z_2, Z_3, Z_4}$ and $f_{A, B}$, i.e., for any $p_1,p_2,p_3,p_4\in \mathbb {R}^3$ with $|p_2 - p_1|= |p_3 - p_2|=|p_4 - p_3| = 1$ we put

2 $$\begin{aligned} {\begin{matrix} f_{P_1,P_2,P_3,P_4}(p_1, p_2, p_3, p_4) = f_{(X_1, Y_1), A_0} ((x_1, y_1), {{\,\textrm{ang}\,}}((x_2, y_2) - (x_1, y_1))) \\ \times f_{Z_1, Z_2, Z_3, Z_4}(z_1, z_2, z_3, z_4) f_{A, B} \bigl (\mathcal {A}(s_2^{(x,y)}, s_1^{(x,y)}), \mathcal {A}(s_3^{(x,y)}, s_2^{(x,y)})\bigr )\,, \end{matrix}} \end{aligned}$$

where $s_i^{(x,y)} = (x_{i+1}, y_{i+1}) - (x_i, y_i)$ for $i=1,2,3$. Note that using Eq. (1), the second factor on the right-hand side of Eq. (2) can be written as product of uni- and bivariate probability densities. Having this in mind, the transition function $f_{P_4 \mid P_1 = p_1, P_2=p_2, P_3=p_3}:\mathbb {R}^3\rightarrow [0,\infty )$ of the Markov chain $\{P_n, n \in \mathbb {Z}\}$ is given by

3 $$\begin{aligned} {\begin{matrix} f_{P_4 | P_1 = p_1, \ldots , P_3 = p_3}(p_4) =&{} f_{Z_3 - Z_2 \mid Z_1 = z_2, Z_2 - Z_1 = z_3 - z_2} (z_4 - z_3) \\ &{}\cdot f_{B \mid A = \mathcal {A}(s_2^{(x,y)}, s_1^{(x,y)})} \bigl ( \mathcal {A}(s_3^{(x,y)}, s_2^{(x,y)}) \bigr ) \end{matrix}} \end{aligned}$$

for any $p_1,p_2,p_3,p_4\in \mathbb {R}^3$ with $|p_2 - p_1|= |p_3 - p_2|=|p_4 - p_3| = c$, where *c* is the constant segment length, $f_{B \mid A = \mathcal {A}(s_2^{(x,y)}, s_1^{(x,y)})}:[-\pi ,\pi ]\rightarrow [0,\infty )$ is the conditional density of *B* given that $A = \mathcal {A}(s_2^{(x,y)}, s_1^{(x,y)})$, and $f_{Z_3 - Z_2 \mid Z_1 = z_2, Z_2 - Z_1 = z_3 - z_2} :[-1, 1] \rightarrow [0, \infty )$ is the conditional density of $Z_3 - Z_2$ given that $Z_1 = z_2$ and $Z_2 - Z_1 = z_3 - z_2$.

#### Selecting parametric families of probability distributions

For utilizing the approach to modeling the transition function $f_{P_4 \mid P_1 = p_1, P_2=p_2, P_3=p_3}$ given in Eq. (3), we have to decide on how to model the tri- and bivariate joint densities $f_{Z_1, Z_2-Z_1, Z_3-Z_2}$ and $f_{A, B}$ of $(Z_1, Z_2-Z_1, Z_3-Z_2)$ and (*A*, *B*), respectively. For this, we separately incorporate knowledge about the measured image data for Sample 1 and Sample 2, respectively, into our model.

**Figure 3:**
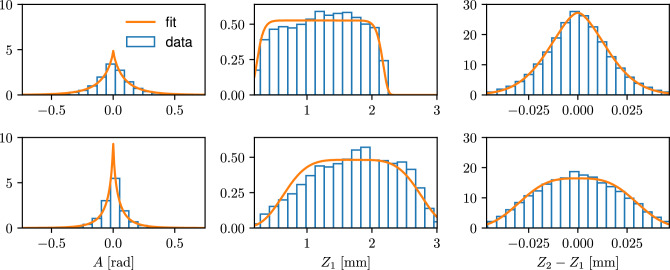
Empirical and fitted distributions of $A, Z_1$ and $Z_2 - Z_1$ for Sample 1 (top) and Sample 2 (bottom). Note that *B* adheres to the same distribution as *A*, and $Z_3 - Z_2$ has the same distribution as $Z_2 - Z_1$.

**Figure 4:**
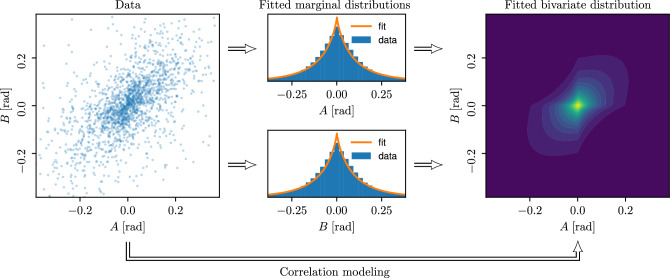
Left: Empirical distribution of (*A*, *B*) for Sample 1, shown as a scatter plot of measured pairs of (*A*, *B*). Center: Fitting generalized normal distributions to the marginal distributions of *A* and *B*, respectively. Right: Modeling the correlation structure of *A* and *B* using a Student’s t copula.

Let $N_F\ge 1$ denote the number of fibers $\left\{ \textsf{F}_i, 1\le i \le N_F \right\}$ with lengths $L_i = |\textsf{F}_i|$, which have been extracted from the corresponding tomographic image, each of which consists of $L_i+1$ vertices $\left\{ p_{i, k}, 1\le k \le L_i+1 \right\}$ for $i\in \left\{ 1,\ldots ,N_F\right\}$. Then, we consider the set of all quadruples $\textsf{Q}_i = \left\{ (p_{i, k}, p_{i, k+1}, p_{i, k+2}, p_{i, k+3}), 1\le k \le L_i - 2 \right\}$ of consecutive vertices along each fiber $\textsf{F}_i$. Furthermore, we take the union set $\textsf{Q} = \cup _{1\le i \le N_F} \textsf{Q}_i$ of all quadruples and, for simplicity, rewrite their indices as $\textsf{Q} = \left\{ (p_{1,j}, p_{2,j}, p_{3,j}, p_{4,j}), 1\le j \le N\right\}$ with $N = \#(\textsf{Q})$, where $\#(Q)$ denotes the cardinality of *Q*. Finally, we compute the corresponding angles $\left\{ (a_j, b_j), 1\le j \le N\right\}$ and *z*-coordinates $\bigl \{\bigl ( z_{1, j}, z_{2, j}, z_{3, j} \bigr ), 1\le j \le N\bigr \}$. Based on this data, we can choose appropriate types of distributions for $f_{Z_1, Z_2-Z_1, Z_3-Z_2}$ and $f_{A, B}$. By examining the tomographic image data considered in this paper, it becomes obvious that in both cases, i.e. for $\left( Z_1, Z_2-Z_1, Z_3-Z_2\right)$ and (*A*, *B*), the components of these random vectors can neither be considered as normally distributed, see Fig. 3, nor independent, see Fig. 4. Thus, we make use of copulas^23,29^ for modeling these distributions, which provide a flexible approach to parametric modeling of multivariate non-Gaussian distributions. In particular for modeling the joint distribution of $\left( Z_1, Z_2-Z_1, Z_3-Z_2\right)$, we employ an R-vine copula^22,23^.

Recall that due to the stationarity of the Markov chain $\{P_n, n \in \mathbb {Z}\}$, only three different marginal distributions need to be modeled. This is, we need to model the marginal distributions of $A, Z_1$ and $Z_2 - Z_1$. Based on two different samples of measured image data, we model all three univariate distributions (of $A, Z_1$ and $Z_2 - Z_1$) by generalized normal distributions^30^. Further details on this parametric family of distributions can be found in the supplementary material. As described there, we chose this family to get a common best fit for both samples. This means that for choosing, e.g., a family of distributions for *A*, we fit a wide variety of parametric families of univariate distributions to the values of *a* obtained for both samples, where we used tools provided by the python package SciPy^31^ and, for each parametric family, compute the likelihood function as a measure for the goodness-of-fit. Then, we choose the distribution family for which the maximum of the likelihood functions among both datasets is largest, see Fig. 3.

The dependence structure of the random vector (*A*, *B*) will be modelled by a bivariate copula. Similar to the procedure described above for choosing univariate marginal distributions, we fit a wide range of parametric copula families to the respective data from both measured samples using the python package pyvinecopulib^32^. Then, we choose the copula which maximizes the goodness-of-fit among both samples with respect to the likelihood function. Thereby, we use the Student’s t-copula to model the dependence structure of (*A*, *B*), see Fig. 4.

For modeling the dependence structure of $(Z_1, Z_2 - Z_1, Z_3 - Z_2)$, we employ an R-vine copula as described in the supplementary material. For this, we need to model three bivariate copulas, namely for the distributions of $(Z_3 - Z_2, Z_1)$ and $(Z_2 - Z_1, Z_1)$ as well as for the conditional distribution of $(Z_3 - Z_2, Z_2 - Z_1)$ conditioning on $Z_1$. Note that, as described in the supplementary material, this choice of an R-vine copula allows for the simulation of $Z_3 - Z_2$, under the condition that the values of $Z_1$ and $Z_2 - Z_1$ are given. Using the same approach as before, we choose to model the dependence structures of $(Z_3 - Z_2, Z_1)$ and $(Z_2 - Z_1, Z_1)$ by Clayton copulas and that of $(Z_3 - Z_2, Z_2 - Z_1)$, conditional on $Z_1$, by a Student’s t-copula.

#### Fitting model parameters

After having selected a parametric family of univariate probability distributions for the (real-valued) random variables $A, Z_1, Z_2 - Z_1$ and parametric copula families for the two-dimensional random vectors (*A*, *B*), $(Z_3 - Z_2, Z_1)$ and $(Z_2 - Z_1, Z_1)$ as well as for the conditional distribution of $(Z_3 - Z_2, Z_2 - Z_1)$ conditioning on $Z_1$, we fit the parameters of these univariate distributions and copulas, again using the data $\left\{ (a_j, b_j), 1 \le j \le N\right\}$ and $\left\{ (z_{1,j}, z_{2,j}, z_{3,j}), 1 \le j \le N\right\}$ described above.

Note that fitting the parameters of the univariate distributions is performed separately for Sample 1 and Sample 2, respectively, by maximum-likelihood estimation provided by SciPy. Figure 3 shows the fitted distributions and the underlying measured data. The parameters of the fitted univariate distributions are given in Table 1.

**Table 1:** Parameters of the fitted univariate distributions. Note that due to symmetry, we would expect a mean (i.e., location) of 0 for the distributions of *A* and $Z_2 - Z_1$, which is in fact (almost) the case for the fitted values.

	Distribution	Sample	Parameters	Location	Scale	Mean	Var
$A~[\text {rad}]$	Gen. normal	1	0.81	0	0.09	0	0.04
		2	0.68	0	0.04	0	0.02
$Z_1~[\text {mm}]$	Gen. normal	1	14.71	1.20	0.98	1.20	0.31
		2	4.38	1.69	1.14	1.69	0.43
$Z_2-Z_1$	Gen. normal	1	1.69	0	0.02	0	0
${[}\text {mm}]$		2	3.12	0	0.03	0	0

The distribution of $Z_1$ essentially dictates the extent of the structures in *z*-direction, where the mean values corresponds to roughly half the thickness of the samples. Note that *B* adheres to the same distribution as *A*, and $Z_3 - Z_2$ has the same distribution as $Z_2 - Z_1$. The units given in the left columns apply also to the location and scale parameters.

Analogously, using pyvinecopulib, the copula parameters are fitted separately for Sample 1 and Sample 2, respectively, based on maximum-likelihood estimation, see Table 2.

**Table 2:** Parameters of the fitted copulas.

	Copula	Sample	Parameters	Rotation
(*A*, *B*)	Student’s t	1	(0.66, 3.15)	0
		2	(0.82, 3.11)	0
$(Z_3 - Z_2, Z_1)$	Clayton	1	0.05	90
		2	0.06	90
$(Z_2 - Z_1, Z_1)$	Clayton	1	0.02	90
		2	0.03	90
$(Z_3 - Z_2, Z_2 - Z_1) | Z_1$	Student’s t	1	(0.93, 2.05)	0
		2	(0.99, 5.00)	0

The results stated in the last three rows are used to build a trivariate R-vine copula for $(Z_1, Z_2-Z_1, Z_3-Z_2)$. Parameters for Student’s t-copulas are given as $(\rho , \nu )$.

#### Simulating the entire fiber system

For simulating the entire fiber system, we fix a bounding cuboid $W = [0, x_{\max }] \times [0, y_{\max }] \times [0, z_{\max }]$ for some $x_{\max },y_{\max },z_{\max }>0$. While we may choose $x_{\max }$ and $y_{\max }$ arbitrarily large, $z_{\max }$ directly relates to the distribution of $Z_1$ which should be theoretically limited to a bounded interval. In practice, however, the best fitting distribution is unbounded and thus, we may choose, e.g., the $99.9 \%$-quantile of this distribution as $z_{\max }$. As a further parameter, we choose the total length $L>0$ of the fiber system within *W*.

Note that we do not explicitly model the lengths of single fibers and assume that all fibers span the whole sampling window *W* which will be guaranteed by the following simulation procedure. This assumption seems largely justified for the considered nonwovens.

To avoid edge-effects, for some $r>0$ we repeatedly simulate fibers in a larger window $\widetilde{W} = [0-r, x_{\max }+r] \times [0-r, y_{\max }+r] \times [0, z_{\max }]$. This means, we start at some initial vertices $p_1, p_2, p_3$ and simulate $\{p_i, i=1,2,\ldots \}$ according to the Markov chain approach described above until the fiber leaves $\widetilde{W}$. Then, we consider those parts of the simulated fiber which lie within the original bounding window *W*, i.e., $\{p_{k_j-1}, \ldots , p_{l_j+1}\}, j \ge 0$, where $1<k_j<l_j$, such that $0 \le x_i \le x_{\max }$ and $0 \le y_i \le y_{\max }$ for all $i \in \{k_j, \ldots , l_j\}$ and $x_i \notin [0, x_{\max }]$ or $y_i \notin [0, y_{\max }]$ for both $i = k_j-1$ and $i = l_j+1$. These parts then only start and end at the boundary of *W*. We stop simulation when the total length of these parts reaches the value of *L*. Note that we did not extend *W* in *z*-direction as the Markov chain $\{Z_n,n\in \mathbb {Z}\}$, which controls the *z*-coordinates, ensures that $z_i$ belongs to the interval $[0, z_{max}]$ for all $i=1,2,\ldots$.

We choose the initial vertices $p_1, p_2, p_3$ by simulating the *z*-coordinates $z_1,z_2,z_3$ of $p_1, p_2, p_3$ using the (known) joint distribution of $Z_1, Z_2 - Z_1, Z_3 - Z_2$. Furthermore, for the first point $p_1=(x_1,y_1,z_1)$, we may simply choose $(x_1, y_1)$ uniformly distributed in $[0, x_{max}] \times [0, y_{max}]$. Then, to generate $(x_2,y_2)$, we simulate the angle between $(x_2, y_2) - (x_1, y_1)$ and the *x*-axis by drawing a sample from the uniform distribution on the interval $[-\pi ,\pi ]$. Finally, to generate $(x_3,y_3)$, we simulate the angle between $(x_3, y_3) - (x_2, y_2)$ and $(x_2, y_2) - (x_1, y_1)$ which is drawn from the known distribution of *A*. Together with the already fixed values of $z_1, z_2, z_3$, we can then easily compute the actual values of $p_2=(x_2, y_2,z_2)$ and $p_3=(x_3, y_3,z_3)$.

For drawing a point $p_{n+1}$ conditional on $p_{n-2}, p_{n-1}, p_n$, we employ the transition function given in Eq. (3). For this, we independently simulate $(x_{n+1}, y_{n+1})$ and $z_{n+1}$ using the fitted distributions described above. Calculating the angle *a* between $(x_n, y_n) - (x_{n-1}, y_{n-1})$ and $(x_{n-1}, y_{n-1}) - (x_{n-2}, y_{n-2})$ and then drawing from *B* conditional on $A = a$ using the probability density $f_{B\mid A = a}$ results in the angle between $(x_{n+1}, y_{n+1}) - (x_n, y_n)$ and $(x_n, y_n) - (x_{n-1}, y_{n-1})$. Furthermore, we draw the value of $z_{n+1} - z_n$ from the probability density $f_{Z_3 - Z_2 \mid Z_1 = z_{n-1}, Z_2 - Z_1 = z_n - z_{n-1}}$ of $Z_3 - Z_2$ conditional on $Z_1 = z_{n-1}$ and $Z_2 - Z_1 = z_n - z_{n-1}$, and then set $z_{n+1}$ accordingly. If $z_{n+1}$ would be outside of the interval $[0, z_{max}]$, we reject it and draw a new value from the conditional distribution of $Z_3 - Z_2$.

Fitting and simulation of the copula-based model described above were implemented in python using, among others, the packages SciPy^31^ and a slightly adapted version of pyvinecopulib^32^.

### Validation measures

Various geometric descriptors of the 3D microstructure of porous media play an important role for transport within the pore space. These descriptors are thus especially well suited to compare the goodness-of-fit between measured image data and model realizations. Assume that we observe a porous material (in our case, a fiber system) in 3D within a bounding cuboid $W=[0, x_{max}] \times [0, y_{max}] \times [0, z_{max}]$ and with some transport direction. In the data at hand, we take the *z*-axis as the transport direction, i.e., we assume that transport through the nonwoven (as opposed to within) is of interest. For reference, we define a “starting plane”, e.g., at $z = 0$ where transport starts and an opposing “end plane”, e.g., at $z = z_{max}$ where transport ends. In the following, we give a short overview over the geometric descriptors considered in the present paper. For more details and further applications, see e.g.^33^.

The geodesic tortuosity is a measure for windedness of transport pathways (within the pore space). For some point $p=(x,y,z)\in W$ with $z=0$ on the starting plane which is contained in the pore space (i.e., not on a fiber), we compute the distance *l* of the shortest path from *p* to the end plane through the pore space using Dijkstra’s algorithm on the voxel grid. By definition, it holds that $l\ge d$, where *d* denotes the distance between starting and end planes, and we call the fraction *l*/*d* the tortuosity of the shortest path starting at *p*. The distribution of these values for randomly selected points *p* is considered as the tortuosity distribution.

The so-called constrictivity is a measure for the strength of bottleneck effects along transport pathways. It is given as a fraction $\frac{r_{min}}{r_{max}} \in [0,1]$. Here, $r_{max}$ is the largest radius such that at least $50 \%$ of pore space can be covered by (potentially overlapping) spheres of radius $r_{max}$. Furthermore, $r_{min}$ is the largest radius such that at least $50 \%$ of pore space can be covered by spheres of radius $r_{min}$ intruding the pore space from the starting plane. This is, a point $p\in W$ inside the pore space is covered by such spheres if there exists a point $p_c$ and a path from the starting plane to $p_c$ such that $|p_c - p| \le r_{min}$ and the distance from any point on said path to the solid phase (i.e., the fibers) is larger than $r_{min}$.

The spherical contact distance for a given point $p\in W$ within the pore space is the shortest distance from *p* to any point within the solid phase (i.e., the fibers). We consider the distribution of these shortest distances for randomly selected points *p*.

## Results

Recall that we fitted the copula-based model, which we consider in this paper, to two different measured datasets of nonwoven structures, and we simulated artificial fiber systems using the fitted models. For comparability, we chose the simulation window, the total length of fibers and fiber diameter to match the values of the measured structures given in the Materials section. Figure 2 illustrates the measured fiber systems along with the simulated structures. Visual comparison of these fiber systems shows a good agreement of the overall morphological properties of measured and simulated fiber systems. In addition, we quantitatively evaluate the agreement between measured and simulated image data, where, among others, we use geometric descriptors of the pore space morphology as explained in the Methods section. In the following, the term “measured” refers to the experimentally measured nonwovens while “simulated” describes the structures obtained from the proposed model. All properties presented in the following are calculated from both types of data in the same way.

**Figure 5:**
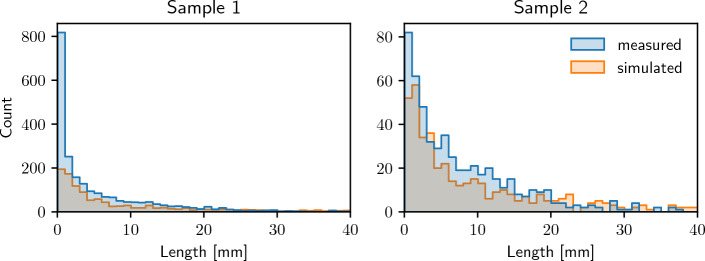
Distribution of fiber lengths, computed from measured image data (blue) and model-based simulations (orange) of Sample 1 (left) and Sample 2 (right).

Considering the bounding window of each of these four datasets, we can compute the specific fiber length, i.e. the total fiber length per unit volume and the porosity of the structure, as shown in Table 3. While these descriptors are closely linked with each other, porosity may not necessarily decrease with increasing number of fibers when fibers are overlapping. Note that, while overlapping fibers are not present in the measured data, we model fibers independently of each other. Thus, we cannot guarantee non-overlapping fibers in the simulations. If non-overlapping fibers are necessary, a suitable displacement algorithm may be applied to the simulated structures. Comparing measured and simulated systems with respect to these geometric descriptors shows a good agreement. Note that the specific fiber lengths of measured and simulated systems are almost identical, which would be expected by definition of the model. The number of fibers is closely linked to the fiber length distribution as shown in Fig. 5. While both the number of fibers and the fiber length distribution match closely between measured and simulated data for Sample 2, fibers tend to be significantly longer in the simulated data when considering Sample 1 which is also reflected in the lower number of simulated fibers, see Table 3. This is probably due to the fact that the measured data of Sample 1 has almost four times the specific fiber length compared to Sample 2 which makes image segmentation challenging and may lead to oversegmentation, i.e., splitting fibers into shorter fragments. Recall that we did not explicitly model the lengths of single fibers and assumed that each fiber spans the whole sampling window.

**Table 3:** Specific fiber length, number of fibers, and porosity, computed from measured image data and model-based simulations of Samples 1 and 2.

Sample	Data	Specific fiber length	Number of fibers	Porosity
		[cm$$^{-2}$$]		
1	Measured	4165	2189	0.987
1	Simulated	4165	1308	0.987
2	Measured	1081	530	0.997
2	Simulated	1084	481	0.997

**Figure 6:**
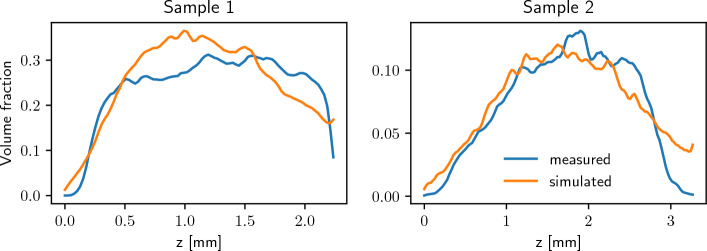
Solid volume fraction for slices at given *z*-position, computed from measured image data (blue) and model-based simulations (orange) of Sample 1 (left) and Sample 2 (right).

Moreover, we computed the solid volume fraction of these structures for slices parallel to the *x*-*y*-plane at different heights $z\in [0,z_{\max }]$. Figure 6 shows the results which we obtained for measured and simulated image data. Especially for Sample 2, they seem to be in good agreement with each other.

**Figure 7:**
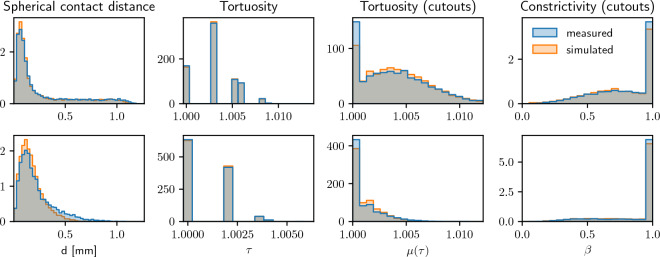
Distribution of geometric descriptors of the pore space morphology, computed from measured image data (blue) and model-based simulations (orange) of Sample 1 (top) and Sample 2 (bottom). Note that as tortuosity is measured on a voxel grid, only finitely many different values of tortuosity can exist which causes the void spaces between the bins in the distributions of tortuosity.

For further validation, we estimated the distribution of the spherical contact distance *d* to the fiber system from a randomly selected location within the pore space, see the first column of Fig. 7. For both Sample 1 and Sample 2, the obtained results show a good agreement between simulated and measured structures. Furthermore, we computed the distribution of geodesic tortuosity $\tau$ of pore space which is a descriptor of the windedness of pathways, see the second column of Fig. 7. Finally, for assessing local heterogeneity, we divided each structure into $20 \times 20 \times 15$ cutouts and computed the mean geodesic tortuosity $\mu (\tau )$ as well as the so-called constrictivity $\beta$ of the pore space on each cutout separately. The third and fourth columns of Fig. 7 show histograms of the values which we obtained for these pore space descriptors. It turned out that the overall agreement between all these distributions is quite good.

## Discussion

In this paper we laid the foundation for data-driven 3D analysis of virtual fiber systems with respect to their microstructure and functionality. In particular, we developed a stochastic 3D model for systems of curved fibers similar to nonwovens, which is fitted to tomographic image data. By systematic variations of model parameters, efficient computer-based scenario analyses can be performed to get a deeper insight how effective properties of this type of functional materials depend on their 3D microstructure.

The modeling approach was based on representing fibers as polygonal tracks which were then modeled by means of third-order Markov chains. By choosing the transition function of these Markov chains to be the product of two (conditional) probability densities, we managed to isolate the influence of intrinsic fiber properties from that of external effects. Modeling these two influencing factors separately yielded comprehensible (conditional) probability densities which can be directly related to different aspects of the geometry of single fibers as well as fiber systems. For a precise description of the underlying multivariate distributions, we chose copulas which minimized the required number of parameters. By construction, the model could be easily adapted to various types of structures which we exemplified by fitting the model to two different measured datasets of nonwovens. Artificial structures simulated by these fitted models resembled the measured datasets with respect to various geometric descriptors of the pore space morphology which are related to macroscopic physical properties of the underlying material and were not used for model fitting. Moreover, the presented model is robust with respect to oversegmentation of the data used for fitting.

As fitting the model and drawing realizations can be performed efficiently with relatively low computational costs, the model is well suited for the development of a framework for virtual materials testing and further investigation of the relationship between microstructure geometry and effective macroscopic properties. This will be investigated in a forthcoming paper^34^.

## Utilized numerical tools

We used the python programming language to implement the model, simulate the structures, compute geometric descriptors and prepare most of the figures for this manuscript. Notably, the following libraries were used: NumPy^35^, SciPy^31^, a slighty modified version of pyvinecopulib^32^, Numba^36^ for accelerated execution and Matplotlib^37^ and seaborn^38^ for creating most of the the figures.

GeoDict^27^ was used to numerically compute the permeability and for 3D rendering of selected structures.

### Supplementary Information

Supplementary Information.

## Data Availability

The data generated and analyzed during this study, i.e., the center-lines used to fit the model and the center-lines of the simulated structures are available from the corresponding author upon reasonable request.

## Code availability

All formulations and algorithms necessary to reproduce the results of this study are described in the “Results” and “Methods” sections and in the Supplementary Information.

## References

[1] Schulz VP, Becker J, Wiegmann A, Mukherjee PP, Wang C-Y (2007). Modeling of two-phase behavior in the gas diffusion medium of PEFCs via full morphology approach. J. Electrochem. Soc..

[2] Kroutilova, J., Maas, M., Mecl, Z., Wagner, T., Klaska, F. & Kasparkova, P. Bulky nonwoven fabric with enhanced compressibility and recovery, (2020). Patent WO2020/103964.

[3] Geerling C, Azimian M, Wiegmann A, Briesen H, Kuhn M (2020). Designing optimally-graded depth filter media using a novel multiscale method. AIChE J..

[4] Huang X, Zhou Q, Liu J, Zhao Y, Zhou W, Deng D (2017). 3D stochastic modeling, simulation and analysis of effective thermal conductivity in fibrous media. Powder Technol..

[5] Schneider M (2017). The sequential addition and migration method to generate representative volume elements for the homogenization of short fiber reinforced plastics. Comput. Mech..

[6] Venkateshan D, Tahir M, Vahedi Tafreshi H, Pourdeyhimi B (2016). Modeling effects of fiber rigidity on thickness and porosity of virtual electrospun mats. Mater. Design.

[7] Abishek S, King A, Mead-Hunter R, Golkarfard V, Heikamp W, Mullins B (2017). Generation and validation of virtual nonwoven, foam and knitted filter (separator/coalescer) geometries for CFD simulations. Sep. Purif. Technol..

[8] Azimian M, Kühnle C, Wiegmann A (2018). Design and optimization of fibrous filter media using lifetime multipass simulations. Chem. Eng. Technol..

[9] Soltani P, Zarrebini M, Laghaei R, Hassanpour A (2017). Prediction of permeability of realistic and virtual layered nonwovens using combined application of X-ray $$\mu$$CT and computer simulation. Chem. Eng. Res. Des..

[10] Wiegmann A, Rief S, Latz A (2007). Computer models of nonwoven geometry and filtration simulation. Filtr. News.

[11] Gaiselmann G, Froning D, Tötzke C, Quick C, Manke I, Lehnert W, Schmidt V (2013). Stochastic 3D modeling of non-woven materials with wet-proofing agent. Int. J. Hydrogen Energy.

[12] Zamel N, Li X, Shen J, Becker J, Wiegmann A (2010). Estimating effective thermal conductivity in carbon paper diffusion media. Chem. Eng. Sci..

[13] Chiverton, J.P., Kao, A., Roldo, M. & Tozzi, G. Volumetric simulation of nano-fibres and 2D SEM and 3D XCT imaging processes. In *Medical Image Understanding and Analysis: 24th Annual Conference, MIUA 2020, Oxford, UK, July 15-17, 2020, Proceedings 24*, pp. 436–445. Springer, (2020).

[14] Kallel H, Joulain K (2022). Design and thermal conductivity of 3D artificial cross-linked random fiber networks. Mater. Design.

[15] Mao N, Russell SJ, Pourdeyhimi B, Russell SJ (2007). Characterisation, testing and modelling of nonwoven fabrics. Handbook of Nonwovens.

[16] Moghadam A, Yousefi SH, Tafreshi HV, Pourdeyhimi B (2019). Characterizing nonwoven materials via realistic microstructural modeling. Sep. Purif. Technol..

[17] Wiegmann A, Bonilla LL, Moscoso M, Platero G, Vega JM (2008). Effective properties of nonwoven textiles from microstructure simulations. Progress in Industrial Mathematics at ECMI 2006.

[18] Chiverton JP, Ige O, Barnett SJ, Parry T (2017). Multiscale Shannon’s entropy modeling of orientation and distance in steel fiber micro-tomography data. IEEE Trans. Image Process..

[19] Herrmann H, Pastorelli E, Kallonen A, Suuronen J-P (2016). Methods for fibre orientation analysis of X-ray tomography images of steel fibre reinforced concrete (SFRC). J. Mater. Sci..

[20] Weber M, Grießer A, Glatt E, Wiegmann A, Schmidt V (2023). Modeling curved fibers by fitting R-vine copulas to their Frenet representations. Microsc. Microanal..

[21] Raftery AE (1985). A model for high-order Markov chains. J. Roy. Stat. Soc.: Ser. B (Methodol.).

[22] Czado C (2019). Analyzing Dependent Data with Vine Copulas.

[23] Joe H (2014). Dependence Modeling with Copulas.

[24] Grießer, A., Westerteiger, R., Wagner, C. & Wiegmann, A. FiberFind: Machine learning-based segmentation and identification of individual fibers in $$\mu$$CT images of fibrous media. In *International Conference on Tomography of Materials & Structures 2019, Cairns, Australia*, (2019).

[25] Grießer A, Westerteiger R, Glatt E, Hagen H, Wiegmann A (2022). Identification and analysis of fibers in ultra-large X-ray scans of nonwoven textiles using deep learning. J. Textile Inst..

[26] Feldkamp LA, Davis LC, Kress JW (1984). Practical cone-beam algorithm. J. Opt. Soc. Am. A-Opt. Image Sci. Vis..

[27] Becker, J., Biebl, F., Boettcher, M., Cheng, L., Frank, F., Glatt, E., Grießer, A., Linden, S., Mosbach, D., Neundorf, A., Wagner, C., Weber, A., Westerteiger, R. & Wiegmann, A. GeoDict Software. 10.30423/release.geodict2023, (2023).

[28] Çiçek Ö, Abdulkadir A, Lienkamp SS, Brox T, Ronneberger O, Ourselin S (2016). 3d u-net: Learning dense volumetric segmentation from sparse annotation. Medical Image Computing and Computer-Assisted Intervention - MICCAI 2016.

[29] Nelsen RB (2006). An Introduction to Copulas.

[30] Nardon M, Pianca P (2009). Simulation techniques for generalized Gaussian densities. J. Stat. Comput. Simul..

[31] Virtanen P, Gommers R, Oliphant TE, Haberland M, Reddy T, Cournapeau D, Burovski E, Peterson P, Weckesser W, Bright J, van der Walt SJ, Brett M, Wilson J, Millman KJ, Mayorov N, Nelson ARJ, Jones E, Kern R, Larson E, Carey CJ, Polat İ, Feng Y, Moore EW, VanderPlas J, Laxalde D, Perktold J, Cimrman R, Henriksen I, Quintero EA, Harris CR, Archibald AM, Ribeiro AH, Pedregosa F, van Mulbregt P (2020). SciPy 1.0 Contributors. SciPy 1.0: Fundamental algorithms for scientific computing in python. Nat. Methods.

[32] Vinecopulib. Vinecopulib/pyvinecopulib: A python library for vine copula models. https://github.com/vinecopulib/pyvinecopulib, (2023). Accessed: 2023-04-20.

[33] Prifling, B. *et al.* Quantifying the impact of 3D pore space morphology on soil gas diffusion in loam and sand. *Trans. Porous Med.*10.1007/s11242-023-01971-z (2023).

[34] Weber, M., Prifling, B., Grießer, A., Glatt, E., Wiegmann, A. & Schmidt, V. Investigating microstructure-property relationships of nonwovens by model-based virtual materials testing. (2023). Working paper (under preparation).

[35] Harris CR, Millman KJ, van der Walt SJ, Gommers R, Virtanen P, Cournapeau D, Wieser E, Taylor J, Berg S, Smith NJ, Kern R, Picus M, Hoyer S, van Kerkwijk MH, Brett M, Haldane A, del Río JF, Wiebe M, Peterson P, Gérard-Marchant P, Sheppard K, Reddy T, Weckesser W, Abbasi H, Gohlke C, Oliphant TE (2020). Array programming with NumPy. Nature.

[36] Lam, S.K., Pitrou, A. & Seibert, S. Numba: A LLVM-based python JIT compiler. In *Proceedings of the Second Workshop on the LLVM Compiler Infrastructure in HPC*, LLVM ’15, New York, NY, USA, (2015). Association for Computing Machinery.

[37] Hunter JD (2007). Matplotlib: A 2D graphics environment. Comput. Sci. Eng..

[38] Waskom ML (2021). Seaborn: statistical data visualization. J. Open Sour. Softw..

