# Supplementary material for: Copula-based modeling and simulation of 3D systems of curved fibers by isolating intrinsic fiber properties and external effects
Source: Sci Rep. 2023 Nov 8;13:19359. doi: 10.1038/s41598-023-46644-5 (PMC10632406; doi:10.1038/s41598-023-46644-5)
Supplement: Supplementary file 1 — Supplementary Information. [file 41598_2023_46644_MOESM1_ESM.pdf]

Supplementary Information

Copula-based modeling and simulation of 3D systems of curved fibers by isolating intrinsic fiber properties and external effects

Matthias Weber,[1,*] Andreas Grießer,[2] Dennis Mosbach,[2] Erik Glatt,[2] Andreas Wiegmann,[2] and Volker Schmidt[1]

[1] Institute of Stochastics, Ulm University, Helmholtzstraße 18, 89069 Ulm, Germany
[2] Math2Market GmbH, Richard-Wagner-Straße 1, D-67655, Kaiserslautern, Germany
[*] corresponding author, matthias.weber@uni-ulm.de

A. Appendix

A.1. Python package for fitting univariate probability distributions

In the context of the present paper, we need to model various univariate data by means of parametric families of probability distributions. The python package SciPy [12] provides tools for using—and especially for fitting—over 100 different distribution families. We can directly exploit this package to fit a specific parametric probability distribution to a given set of observations. Moreover, to choose the best suited distribution for the dataset, we fit all distribution families available in SciPy to the data, where we use the likelihood function as a measure for the goodness-of-fit for each fitted distribution. Based on these values, we choose the best fitting distribution for the given dataset, i.e., we choose the distribution family for which the maximum of the likelihood functions is largest. By this approach, we decide which parametric families we use to model the marginal distributions of the random vectors comprised in the copula-based model considered in the present paper.

However, note that we base this decision on two datasets. While we may not assume that a property follows the same type of distribution for both datasets, we would like our model to be able to compare the different morphologies of both datasets with each other. Thus, we would like to find a distribution family which (using different parameters) captures the empirical distribution of the considered property for both datasets.

It turned out that for all univariate data considered in this paper, the generalized normal distribution [7] provides the best fit. This is a parametric family of univariate distributions with parameters $\mu \in \mathbb{R}, \alpha, \beta > 0$, whose density $f_{\mu,\alpha,\beta} : \mathbb{R} \to [0, \infty)$ is given by

$$f_{\mu,\alpha,\beta}(x) = \frac{\alpha, \beta}{2\alpha\Gamma(1/\beta)} \exp\left(-(|x-\mu|/\alpha)^{\beta}\right), \qquad \text{for all } x \in \mathbb{R},$$

where μ, α and β are known as location, scale and shape parameters, respectively, and $\Gamma : [0, \infty) \to [0, \infty)$ denote the gamma function. For our purposes, we need to determine the distribution function and quantile function of the generalized normal distribution which are computed numerically using the python package SciPy [12].

A.2. Sklar's representation formula for multivariate distributions

For modeling multivariate probability distributions, different approaches exist. While some univariate distribution families like normal, Student's t [4], or von Mises [8] distributions have multivariate counterparts with parametric probability densities, parametrically describing the multivariate distribution of arbitrary random vectors $(X_1, \ldots, X_d)$ for any given integer $d \geq 2$ requires more advanced theory. This is indeed the case for the multivariate distributions considered in the present paper. For modeling such multivariate probability distributions, so-called copulas can be exploited [6, 9]. Using copulas, the univariate marginal distributions of a random vector can be chosen separately and fitted directly to the data. Then, in a second step, the dependence structure of the data can be modeled by means of a copula, which turns the marginal distributions into the desired multivariate distribution.

A d-variate copula is a d-variate (cumulative) distribution function $C\colon [0,1]^d \to [0,1]$ with uniform marginal distributions on the unit interval $[0,1]$. According to Sklar's representation formula [6, 9], for any d-variate distribution function $F\colon \mathbb{R}^d \to [0,1]$ with marginal distribution functions $F_1, \ldots, F_d\colon \mathbb{R} \to [0,1]$ a d-variate copula $C\colon [0,1]^d \to [0,1]$ exists such that

$$F(x_1, \ldots, x_d) = C(F_1(x_1), \ldots, F_d(x_d)) \qquad \text{for all } x_1, \ldots, x_d \in \mathbb{R}\,. \tag{1}$$

Note that in the case when the joint distribution function $F : [0,1]^d \to [0,1]$ of a certain random vector $(X_1, \ldots, X_d)$ has a probability density $f : \mathbb{R}^d \to [0, \infty)$, this leads to an equivalent version of Eq. (1) in terms of the probability density $c : [0,1]^d \to [0, \infty)$

corresponding to C. Namely, then it holds that

$$f(x_1,\ldots,x_d) = c(F_1(x_1),\ldots,F_d(x_d))\, f_1(x_1)\ldots f_d(x_d)) \quad \text{for all } x_1,\ldots,x_d \in \mathbb{R}\,, \tag{2}$$

where $f_i : \mathbb{R} \to [0,\infty)$ is the density corresponding to F_i for $i \in \{1,\ldots,d\}$.

Furthermore, the transformed components $F_1(X_1),\ldots,F_d(X_d)$ of the random vector $(X_1,\ldots,X_d)$ are then uniformly distributed on $[0,1]$. Thus, after fitting the marginal density $f_i : \mathbb{R} \to [0,\infty)$ to a dataset $(x_i^{(1)},\ldots,x_i^{(N)}) \in \mathbb{R}^N$ for each $i = 1,\ldots,d$ as described above, and after computing the cumulative distribution functions $F_1,\ldots,F_d$, which is typically done by numerical integration of $f_1,\ldots,f_d$, respectively, the transformed data $(F_1(x_1^{(j)}),\ldots,F_d(x_d^{(j)})), 1 \leq j \leq N$, can be used to fit a parametric probability density $c : [0,1]^d \to [0,\infty)$.

Various parametric models for copulas are considered in the literature [6, 9], many of which are so-called Archimedean copulas $C : [0,1]^d \to [0,1]$ with

$$C(u_1,\ldots,u_d) = \psi^{-1}(\psi(u_1) + \ldots + \psi(u_d)) \qquad \text{for all } u_1,\ldots,u_d \in [0,1],$$

where the so-called generator function $\psi\colon [0,1] \to [0,\infty)$ is a continuous, strictly decreasing convex function such that $\psi(1) = 0$ and ψ^{-1} is the pseudo-inverse of ψ. Examples of Archimedean copulas which we use in the present paper include the Clayton copula with generator function $\psi_\theta : [0,1] \to (0,\infty)$ given by

$$\psi_\theta(u) = \frac{u^{-\theta} - 1}{\theta} \qquad \text{for all } u \in [0,1]$$

and some parameter $\theta \in [-1,\infty) \setminus \{0\}$.

Furthermore, some copulas correspond to known multivariate probability distributions. This is due to the fact that for any d-variate cumulative distribution function $F : \mathbb{R}^d \to [0,1]$ as considered above, it can be easily shown that by the function $C : [0,1]^d \to [0,1]$ with

$$C(u_1,\ldots,u_d) = F(F_1^{-1}(u_1),\ldots,F_d^{-1}(u_d)) \qquad \text{for all } u_1,\ldots,u_d \in [0,1]$$

a d-variate copula is given which can be used to model F. Commonly used copulas derived in this way include the Student's t-copula which is based on the multivariate Student's t-distribution. In the 2-dimensional case, this distribution is defined by its probability density $f_{\nu,\mu,\Sigma} : \mathbb{R}^2 \to [0,\infty)$ function with

$$f_{\nu,\mu,\Sigma}(x) = \frac{\Gamma\left[(\nu+2)/2\right]}{\Gamma(\nu/2)\nu\pi\left|\Sigma\right|^{1/2}} \left[1 + \frac{1}{\nu}(x-\mu)^\top \Sigma^{-1}(x-\mu)\right]^{-(\nu+2)/2} \qquad \text{for all } x \in \mathbb{R}^2,$$

for some degree of freedom $\nu > 0$, location vector $\mu \in \mathbb{R}^2$ and positive-definite scale matrix $\Sigma \in \mathbb{R}^{2\times 2}$. Note that for the definition of the copula, μ and the diagonal elements of Σ are irrelevant and can be set to 0 and 1, respectively. This leaves the bivariate Student's t-copula with two parameters: $\nu > 0$ for the degree of freedom, and $\rho > 0$ for the non-diagonal entries of Σ of the underlying bivariate t-distribution.

A.3. R-vine copulas

While especially Archimedean copulas can easily be used for any dimension $d \geq 2$, many multivariate distributions in higher dimensions can not be expressed by means of Archimedean copulas. Then, so-called R-vine copulas provide a flexible approach which can be used for modeling arbitrary multivariate distributions. A general theory and some applications of R-vine copulas can be found, e.g., in [1, 2, 3, 6]. In the present paper, we use an approach similar to R-vine copulas to model the trivariate probability densities which have been used in the model.

Let $X = (X_1, X_2, X_3)$ be a three-dimensional random vector with joint probability density $f\colon \mathbb{R}^3 \to [0,\infty)$, marginal densities $f_1, f_2, f_3 : \mathbb{R} \to [0,\infty)$ and corresponding cumulative distribution functions $F_1, F_2, F_3\colon \mathbb{R} \to [0,1]$. Then, using Eq. (2) and Bayes' theorem of probability calculus, the trivariate density f can be written as

$$\begin{aligned} f(x_1,x_2,x_3) = {} & c_{2,3|X_1=x_1}(F_{2|X_1=x_1}(x_2), F_{3|X_1=x_1}(x_3)) \\ & \times c_{2,1}(F_2(x_2), F_1(x_1))\, c_{3,1}(F_3(x_3), F_1(x_1)) \\ & \times f_1(x_1) f_2(x_2) f_3(x_3)\,, \end{aligned} \tag{3}$$

for all $(x_1,x_2,x_3) \in \mathbb{R}^3$ such that $f(x_1,x_2,x_3) > 0$. Here, $c_{i,1}$ is the copula density corresponding to the distribution of (X_i, X_1) for $i = 2,3$, and $c_{2,3|X_1=x_1}$ is that corresponding to the distribution of (X_2, X_3) conditional on $X_1 = x_1$. Furthermore, $F_{i|X_1=x_1}$ denotes the distribution function of X_i conditional on $X_1 = x_1$ for $i = 2,3$.

For computational feasibility, one generally assumes that the bivariate copula density $c_{2,3|X_1=x_1}$ does not depend on the specific value x_1 of X_1, denoting it by $c_{2,3|1}$, see [5]. We also remark that the order of the entries X_1, X_2, X_3 of the random vector (X_1, X_2, X_3) can be changed, i.e., on the right-hand side of Eq. (3) one can condition on $X_2 = x_2$ or $X_3 = x_3$, instead of conditioning on $X_1 = x_1$. In this way, the right-hand side of Eq. (3) can be adjusted to suit the needs for further use of this copula representation formula.

A.4. Fitting of copula densities

For fitting a multivariate probability density $f : \mathbb{R}^d \to [0,\infty)$ to data, we need to fit the copula density $c : [0,1]^d \to [0,1]$ appearing on the right-hand side of Eq. (2), where we exploit the C-library vinecopulib and its python bindings (pyvinecopulib) [11] for choosing an appropriate copula model and calibrating it to given data. For a large variety of parametric copula families, this package provides tools for data fitting, simulation and further purposes.

For $d = 2$, similar to choosing marginal distributions as described above, we choose bivariate copula families which perform equally well for two different datasets. More

precisely, for fitting the copula density $c : [0,1]^2 \to [0,1]$, after choosing appropriate marginal distribution functions F_1 and F_2 for a dataset $(x_1^{(1)}, x_2^{(1)}), \ldots, (x_1^{(N)}, x_2^{(N)})$ and transforming the data into the dataset $(u_1^{(1)}, u_2^{(1)}), \ldots, (u_1^{(N)}, u_2^{(N)})$, where $u_j^{(i)} = F_j(x_j^{(i)})$ for $i = 1, \ldots, N$ and $j = 1, 2$, we use the tools provided by vinecopulib to fit various copula models to $(u_1^{(1)}, u_2^{(1)}), \ldots, (u_1^{(N)}, u_2^{(N)})$. In particular, for each dataset extracted from Sample 1 and Sample 2, respectively, and for each bivariate copula model provided by vinecopulib, we compute the log-likelihood function and finally choose the copula such that the minimum of the maximum log-likelihood among both datasets is maximized. Similar to the method explained above for choosing the marginal distributions, this ensures that the behavior of both datasets can be captured equally well using the same copula, albeit with different parameters.

Note that also in the three-dimensional case ($d = 3$), for fitting a trivariate probability density $f : \mathbb{R}^3 \to [0, \infty)$ to data, we only need to fit bivariate copulas, using the representation formula given in Eq. (3). For example, for fitting the (unconditional) copula density $c_{2,1}$ appearing on the right-hand side of Eq. (3), after choosing appropriate marginal distribution functions F_2 and F_1 for a dataset $(x_2^{(1)}, x_1^{(1)}), \ldots, (x_2^{(N)}, x_1^{(N)})$ and transforming the data into the dataset $(u_2^{(1)}, u_1^{(1)}), \ldots, (u_2^{(N)}, u_1^{(N)})$, where $u_j^{(i)} = F_j(x_j^{(i)})$ for $i = 1, \ldots, N$ and $j = 1, 2$, where we again use the tools provided by vinecopulib to fit various copulas, but now to the dataset $(u_2^{(1)}, u_1^{(1)}), \ldots, (u_2^{(N)}, u_1^{(N)})$. Moreover, as in the two-dimensional case described above, for each dataset extracted from Sample 1 and Sample 2, respectively, and for each bivariate copula model provided by vinecopulib, we compute the log-likelihood function and choose the copula such that the minimum of the maximum log-likelihood among both datasets is maximized. The other (unconditional) copula density $c_{3,1}$ on the right-hand side of Eq. (3) is fitted to data in the same way. For fitting the (conditional) copula density $c_{2,3|1}$ on the right-hand side of Eq. (3), we consider the dataset $(x_1^{(1)}, x_2^{(1)}, x_3^{(1)}), \ldots, (x_1^{(N)}, x_2^{(N)}, x_3^{(N)})$ and transform it into the dataset $(u_1^{(1)}, u_2^{(1)}, u_3^{(1)}), \ldots, (u_1^{(N)}, u_2^{(N)}, u_3^{(N)})$,as described above for the two-dimensional case. Then, using so-called h-functions, we further transform the latter dataset into the dataset $(\widetilde{u}_2^{(1)}, \widetilde{u}_3^{(1)}), \ldots, (\widetilde{u}_2^{(N)}, \widetilde{u}_3^{(N)})$, where $\widetilde{u}_j^{(i)} = h_{j,1}(u_j^{(i)}, u_1^{(i)})$ for $i = 1, \ldots, N$ and $j = 2, 3$ and $h_{j,1}(u_j, u_1) = \mathbb{P}(U_j \le u_j \mid U_1 = u_1)$ for $j = 2, 3$. Here, (U_j, U_1) denotes a two-dimensional random vector with (joint) probability density $c_{j,1}$ for $j = 2, 3$. Finally, the copula density $c_{2,3|1}$ is fitted to the dataset $(\widetilde{u}_2^{(1)}, \widetilde{u}_3^{(1)}), \ldots, (\widetilde{u}_2^{(N)}, \widetilde{u}_3^{(N)})$, as described above.

A.5. Drawing samples from conditional probability densities

For simulation purposes, we need methods to draw samples from (conditional) distributions modeled by a copula approach as given in Eqs. (1) and (3), where we use the tools provided by vinecopulib [11].

For conditional sampling, we first consider the two-dimensional case where, according to Eq. (1), the joint distribution function of (X_1, X_2) is given by the marginal distrinution functions $F_1\colon \mathbb{R} \to [0,1]$ and $F_2\colon \mathbb{R} \to [0,1]$ of X_1 and X_2, respectively, and the copula $C\colon [0,1]^2 \to [0,1]$ describing the joint distribution function of $U_1 = F_1(X_1)$ and $U_2 = F_2(X_2)$. Drawing a value x_2 from X_2 conditional on $X_1 = x_1$ can be performed by drawing a value u_2 from U_2 conditional on $U_1 = F_1(x_1)$ and setting $x_2 = F_2^{-1}(u_2)$. For this, we employ the inverse of the so-called first h-function $h\colon [0,1]^2 \to [0,1]$ corresponding to the copula C which is defined as

$$h(u_1, u_2) = \mathbb{P}(U_2 \leq u_2 \mid U_1 = u_1) \qquad \text{for any } u_1, u_2 \in [0,1], \tag{4}$$

where the inverse $h^{-1} : [0,1]^2 \to [0,1]$ of this function is computed with respect to the second argument, i.e., $h^{-1}(u_1, v)$ is the v-quantile of the distribution of U_2 conditional on $U_1 = u_1$. For some copulas, analytical methods to evaluate the h-functions and their inverse exist while for others, numerical methods can be used. For the copulas used in our model, they are implemented in vinecopulib [11]. Now, we can apply the inversion method for simulating from the distribution of U_2 conditional on $U_1 = u_1$ [10]. This is, drawing a sample $x_2 \in \mathbb{R}$ from X_2 conditional on $X_1 = x_1$ amounts to drawing a sample $v \in [0,1]$ from the (standard) uniform distribution on the unit interval $[0,1]$ and computing

$$x_2 = F_2^{-1}(h^{-1}(F_1(x_1), v))\,.$$

We use this approach to sample the angle B conditional on the value of A.

In the three-dimensional case, we need to sample a random variable X_3 conditional on two random variables X_1 and X_2, where the joint density of (X_1, X_2, X_3) is given by the copula representation as stated in Eq. (3).

For doing this, we use the so-called Rosenblatt transform [6] which, in the three-dimensional case, maps a sample (x_1, x_2, x_3) of (X_1, X_2, X_3) onto a sample (r_1, r_2, r_3) of (R_1, R_2, R_3), where R_1, R_2, R_3 are independent random variables which are uniformly distributed on $[0,1]$ such that

$$\begin{aligned} r_1 &= F_1(x_1), \\ r_2 &= F_{2|X_1=x_1}(x_2), \\ r_3 &= F_{3|X_1=x_1,X_2=x_2}(x_3), \end{aligned}$$

where $F_1\colon \mathbb{R} \to [0,1]$ denotes the cumulative distribution function of X_1 and $F_{2|X_1=x_1}$ and $F_{3|X_1=x_1,X_2=x_2}$ denote the conditional distribution functions of X_2 conditional on X_1, and X_3 conditional on X_1 and X_2, respectively. When the corresponding densities are non-zero, the inverse Rosenblatt transform can be obtained by

$$\begin{aligned} x_1 &= F_1^{-1}(r_1), \\ x_2 &= F_{2|X_1=x_1}^{-1}(r_2), \\ x_3 &= F_{3|X_1=x_1,X_2=x_2}^{-1}(r_3). \end{aligned} \tag{5}$$

As R_1, R_2, R_3 are independent, drawing from X_3 conditional on $X_1 = x_1$ and $X_2 = x_2$ can be performed by drawing r_3 from the uniform distribution on $[0,1]$ and then computing x_3 using the inverse Rosenblatt transform, see Eq. (5). As the joint density of (X_1, X_2, X_3) can be expressed by the pair-copula representation as stated in Eq. (3), the expression for x_3 given above can be written as

$$x_3 = F_3^{-1}(h_{1,3}^{-1}(r_1, h_{2,3|1}^{-1}(r_2, r_3)), \tag{6}$$

where $r_1 = F_1(x_1)$, $r_2 = h_{1,2}(r_1, F_2(x_2))$ and $h_{1,3}, h_{1,2}, h_{2,3|1} \colon [0,1]^2 \to [0,1]$ are h-functions similar to Eq. (4) given by

$$\begin{aligned} h_{1,2}(u_1, u_2) &= \mathbb{P}(U_2 \leq u_2 \mid U_1 = u_1), \\ h_{1,3}(u_1, u_3) &= \mathbb{P}(V_3 \leq u_3 \mid V_1 = u_1), \\ h_{2,3|1}(u_2, u_3) &= \mathbb{P}(W_3 \leq u_3 \mid W_2 = u_2) \end{aligned}$$

for $u_1, u_2, u_3 \in [0,1]$. Here, the random vectors (U_2, U_1), (V_3, V_1) and (W_2, W_3) are distributed according to the copula densities $c_{2,1}$, $c_{3,1}$, $c_{2,3|1}$ appearing in Eq. (3), respectively. As in the two-dimensional case, the inverse h-functions in Eq. (6) are computed with respect to the second argument and are implemented in pyvinecopulib [11]. We use this approach for simulating $Z_3 - Z_2$ conditional on the values of $Z_2 - Z_1$ and Z_1.

References

[1] K.-M. Aigner, P. Schaumann, F. von Loeper, A. Martin, V. Schmidt, and F. Liers. Robust DC optimal power flow with modeling of solar power supply uncertainty via R-vine copulas. *Optimization and Engineering*, 24:1951–1982, 2023.

[2] C. Czado. *Analyzing Dependent Data with Vine Copulas.* Springer, 2019.

[3] O. Furat, T. Kirstein, T. Leißner, K. Bachmann, J. Gutzmer, U. A. Peuker, and V. Schmidt. Multidimensional characterization of particle morphology and mineralogical composition using CT data and R-vine copulas. *Minerals Engineering*, submitted, https://doi.org/10.48550/arXiv.2301.07587, 2023.

[4] A. Genz and F. Bretz. *Computation of Multivariate Normal and t Probabilities*, volume 195. Springer Science & Business Media, 2009.

[5] I. H. Haff, K. Aas, and A. Frigessi. On the simplified pair-copula construction–simply useful or too simplistic. *Journal of Multivariate Analysis*, 101(5):1296–1310, 2010.

[6] H. Joe. *Dependence Modeling with Copulas.* Chapman and Hall/CRC, 2014.

[7] M. Nardon and P. Pianca. Simulation techniques for generalized Gaussian densities. *Journal of Statistical Computation and Simulation*, 79(11):1317–1329, 2009.

[8] A. Navarro, J. Frellsen, and R. Turner. The multivariate generalised von Mises distribution: Inference and applications. In *Proceedings of the AAAI Conference on Artificial Intelligence*, volume 31, pages 2394–2400, 2017.

[9] R. B. Nelsen. *An Introduction to Copulas.* Springer, 2006.

[10] C. P. Robert, G. Casella, and G. Casella. *Monte Carlo Statistical Methods*, volume 2. Springer, 1999.

[11] Vinecopulib. Vinecopulib/pyvinecopulib: A python library for vine copula models. `https://github.com/vinecopulib/pyvinecopulib`, 2023. Accessed: 2023-04-20.

[12] P. Virtanen, R. Gommers, T. E. Oliphant, M. Haberland, T. Reddy, D. Cournapeau, E. Burovski, P. Peterson, W. Weckesser, J. Bright, S. J. van der Walt, M. Brett, J. Wilson, K. J. Millman, N. Mayorov, A. R. J. Nelson, E. Jones, R. Kern, E. Larson, C. J. Carey, İ. Polat, Y. Feng, E. W. Moore, J. VanderPlas, D. Laxalde, J. Perktold, R. Cimrman, I. Henriksen, E. A. Quintero, C. R. Harris, A. M. Archibald, A. H. Ribeiro, F. Pedregosa, P. van Mulbregt, and SciPy 1.0 Contributors. SciPy 1.0: Fundamental algorithms for scientific computing in python. *Nature Methods*, 17:261–272, 2020.
